# Yahtzee: An Anonymized Group Level Matching Procedure

**DOI:** 10.1371/journal.pone.0055760

**Published:** 2013-02-05

**Authors:** Jason J. Jones, Robert M. Bond, Christopher J. Fariss, Jaime E. Settle, Adam D. I. Kramer, Cameron Marlow, James H. Fowler

**Affiliations:** 1 Political Science Department and Medical Genetics Division, University of California San Diego, La Jolla, California, United States of America; 2 Political Science Department, University of California San Diego, La Jolla, California, United States of America; 3 Government Department, College of William & Mary, Williamsburg, Virginia, United States of America; 4 Data/Science, Facebook, Inc., Palo Alto, California, United States of America; Cinvestav-Merida, Mexico

## Abstract

Researchers often face the problem of needing to protect the privacy of subjects while also needing to integrate data that contains personal information from diverse data sources. The advent of computational social science and the enormous amount of data about people that is being collected makes protecting the privacy of research subjects ever more important. However, strict privacy procedures can hinder the process of joining diverse sources of data that contain information about specific individual behaviors. In this paper we present a procedure to keep information about specific individuals from being “leaked” or shared in either direction between two sources of data without need of a trusted third party. To achieve this goal, we randomly assign individuals to anonymous groups before combining the anonymized information between the two sources of data. We refer to this method as the Yahtzee procedure, and show that it performs as predicted by theoretical analysis when we apply it to data from Facebook and public voter records.

## Introduction

Computational social science is an emergent field of inquiry that promises to revolutionize the way we study and understand human behavior [1], [2]. Unfortunately, obstacles exist that hamper analysis of the large-scale dynamic datasets that are now available [2]. One problem is that companies (such as Google, Facebook, or cell phone providers, for example) are often reluctant to share with external researchers data obtained from their clients. When they do allow access to the data, it is often in an aggregated or anonymized format designed to protect the identities of their users. While this approach has led to a variety of collaborative research projects [3], once identifying information is removed from the data it cannot be combined with other data sources, limiting the type and scope of research that can be performed with such data.

This article presents a procedure designed to address this issue by keeping information about specific individuals private while still allowing researchers to combine multiple sources of information in such a way that they can still make inferences about the relationships between variables across sets of data. In this way, the method developed in this paper ensures anonymity *ex post* by adding uncertainty about an individual's response after data collection through a group-level matching procedure. The method is conceptually similar but procedurally distinct from other techniques designed to ensure respondent anonymity *a priori* at the time of data collection [4]–[7].

To address these issues we have developed a method to anonymously match group level information. This method is different from other “privacy preserving” machine learning techniques, in that our process joins distinct information about individuals from at least two sources [8] whereas “privacy preserving” machine learning techniques pool similar data from multiple sources. The “Yahtzee” procedure also does not rely on a third party function to join the constituent datasets as some other procedures do [9]. A review of other computational methods for ensuring case level anonymity can be found elsewhere [10]. The advantage of the “Yahtzee” procedure is that no party ever needs to know certain information about a specific individual because this information is anonymized prior to merging the constituent datasets. The Yahtzee procedure can therefore be used to preprocess a dataset before it is ever sent to another organization for analysis. Thus, multiple data collecting entities can use this procedure to anonymize their data before combining that data with other sources for joint analysis.

We developed this method in order to join information from public voting records with data we are analyzing through a collaborative research project with Facebook [11]. Although our human subjects protocol approved by the Institutional Review Board at the University of California, San Diego, allows us to perform one-to-one matching of Facebook data and voter records, Facebook asked us to design a procedure that would better protect the privacy of its users. Thus, in order to study the voting behavior of users, it was necessary to devise a process for matching users to their publicly available voting records without identifying the behavior of specific users. In this process, we wanted to be sure that information about specific individuals was not “leaked” or shared in either direction. Our goal was to avoid connecting any specific Facebook user's voting behavior to Facebook's database of information about a given user. To achieve this goal, we devised a group-level matching procedure that repeatedly randomly assigned users to groups, allowing us to know a user's turnout decision with a given probability, as we describe below. The procedure ensures that the group level value never implicates an individual in either dataset. This would only be possible if both datasets were exactly the same.

In the next section of this article we describe the method and then follow this with a discussion of our application that links publicly available voter records with Facebook data. We then validate the method and close with a discussion of the usefulness of the method for computational social science research generally.

## Materials and Methods

### Group-Level Anonymous Matching

In this section of the paper we describe the Yahtzee method generally and then apply it to datasets that contain voter registration data, which we anonymously combine with Facebook data. It is worth mentioning early and often that the method ensures that only group-level data is passed from one dataset to the other. Individual level data is never merged between datasets. This is accomplished by following several steps that can be applied to virtually any dataset.

Our goal was to match publicly available validated voting records to the records of Facebook users, while protecting the privacy of users' information by not identifying the behavior of individual users. In the United States, turnout behavior is a matter of public record (note that “turnout” refers to whether an individual voted, not *how* an individual voted). However, states vary in how they keep these records and the procedures and costs associated with accessing them. To choose which states to validate, we identified those that provided (for research purposes) first names, last names, and full birth dates in publicly available voting records. From these, we chose a set that minimized the cost per individual voting record. Of these states, the cost of voting records varied from $0 to $1500 per state. We excluded records from Texas because they systematically excluded some individuals from their voting records (specifically, they did not report on the voting behavior of people that had abstained in the four prior elections). The resulting list of 13 states included Arkansas, California, Connecticut, Florida, Kansas, Kentucky, Missouri, Nevada, New Jersey, New York, Oklahoma, Pennsylvania, and Rhode Island. In our application, the “origin” dataset was a state's voter record file and the “destination” dataset was the Facebook data.

We make a distinction between the origin and destination dataset for ease of exposition but in practice the procedure is conducted on both datasets and the group level information can be shared by the holders of either of the original datasets. However, to increase the match rate and statistical efficiency of the analysis, the holder of the larger dataset should match groups generated from the smaller dataset. We discuss this issue when describing the overall match rate when we apply the method. In short, a higher match rate is achieved by sending the group level data to the holder of the larger dataset for analysis.

We begin by removing duplicate rows from the origin dataset (state voter files in our application). In our case, these are individuals who have the same first name, last name and date of birth. In the first step of the procedure we produce a unique identifier for each individual in each of the datasets we wish to merge. To do so we take identifying information that is common across those datasets (in our case, first name, last name, and date of birth), concatenate them together and generate an encrypted one-way hash. The hash is a numeric hexadecimal value which we modify to create a group ID.

When creating the user specific hexadecimal ID, it is also necessary to use a different random number seed and a “salt.” A salt is a character string that is added to the end of the unique identifier in each round of the procedure. In each round the salt is changed so that the hashing procedure produces a new, unique hash for the given individual. The salt allows us to generate multiple hashes per user without getting the same hash every iteration of the process. This ensures that user information cannot be unhashed given knowledge of individuals in a specific dataset. An example dataset is displayed in Table 1 which demonstrates the concatenation of identifying information and the “salt.” In our application, we “salt” the concatenated values by adding 4 randomly generated characters to the end of each string prior to using the hashing algorithm.

**Table 1:** Hash Example.

	first name	last name	date of birth	Salt	concatenated value to hash	last 7 hash digits
1	Jason	Jones	11/07/1977	XKCD	JASONJONES19771107XKCD	b815d72
2	Robert	Bond	10/2/1983	XKCD	ROBERTBOND19831021XKCD	3863afe
3	Christopher	Fariss	11/18/1981	XKCD	CHRISTOPHERFARISS19811118XKCD	e0df6f8
4	Jaime	Settle	7/5/1985	XKCD	JAIMESETTLE19850705XKCD	c2e47b1
5	Adam	Kramer	1/24/1981	XKCD	ADAMKRAMER19810124XKCD	947407f
6	Cameron	Marlow	3/28/1977	XKCD	CAMERONMARLOW19770328XKCD	e4b91f9
7	James	Fowler	2/18/1970	XKCD	JAMESFOWLER19700218XKCD	46221bc
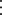	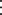	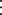	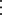	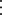	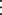	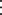
*N*						

Example hash of first name, last name and date of birth. The last 7 digits of the SHA-256 hash value are kept and the rest of the hash discarded because of memory limitations. The 7 digit hash is a numeric hexadecimal value. For step 1, each round of the Yahtzee procedure begins with the hashing of the datasets using a new salt. The “salt” allows us to generate multiple hashes without getting the same hash every round. Next, the hash is divided by the value 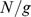, where 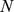 is the number of individuals in the dataset and 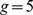 was chosen arbitrarily. The remainder of this calculation is recorded as the group ID. Records are then placed into groups of various sizes based on this group ID. On average the groups should contain 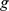 records. Next the frequency of some behavior of interest - in our case voting - is recorded for each group ID. In subsequent steps, a group ID is generated using the identical process on a second dataset. In the second dataset, the frequency of the behavior of interest is assigned to each record based on its group ID. In some cases, the same record is in both datasets, and its contribution to the value assigned to the group in the origin dataset will be transferred to the group in the destination dataset. However, individual records are never matched. We can be sure that identical records in both datasets will be assigned the same group ID, but we can never be sure for any one record if a true match exists in the other dataset or just records that hash to values with the same remainder after dividing by 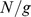.

Next, the hash is divided by the value 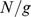, where 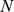 is the number of individuals in the dataset and 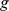 is the size of the groups that the researcher wishes to use. We have chosen to set 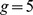, but this is an arbitrary decision that can be changed. Each individual, 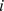, is then assigned a group ID that is equal to the *remainder* of 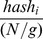.

We then place individuals into groups based on this ID. This step generates groups of various sizes, but on average, groups will be of size 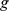. Sampling variation causes some groups to contain more or fewer than 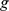 respondents; these groups should be discarded because knowledge of the group size is necessary for statistical inference later in the procedure. On average, though, the groups should contain 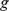 records. It should be apparent as well that smaller values of 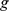 lead to increased uncertainty about the behavior rate of the group for each round of the procedure.

We record the frequency of the behavior of interest for each group ID. In our application this behavior is voter turnout. We also record the group ID.

Next, we generate a group ID using an identical process on the second, destination dataset (Facebook data in our application). We then “match” the two data sets based on the recorded group IDs we create. A “match” is defined as a row in both files that has the same group ID. Individuals who have the same starting values (in the example above, the same name and birthdate) will have the same hash value. Individuals with the same hash value will be assigned to the same group.

In some cases, the same record exists in both datasets, and its contribution to the value assigned to the group in the origin dataset will be transferred to the group in the destination dataset. However, we can never be sure if an individual in one of the datasets is also in the other. We can therefore be sure that identical records in both datasets will be assigned the same group ID but we never know for which records this is the case.

Importantly, we can never be certain about the behavior of an individual in either group because we are never certain if the groups in the two datasets contain the same individuals. We know only that if the same record is in both datasets it will be assigned the same group ID but we can never be certain if the individuals in the two groups are the same.

Moreover, because we never transfer individual-level data we can never be certain about an individual-level value. Records in the destination data are always assigned values, but we are uncertain whether those values are assigned due to a “match” or if they are assigned due to a shared group ID without a true “match.” That is, we are never certain if individuals are in both groups and the group-level matches therefore lead to the assignment of values to individual level records that would not otherwise be matched.

It is therefore worth repeating that the group level value never implicates an individual in either dataset. This would only be possible if both datasets were exactly the same, which would allow us to be certain that the individuals in groups with the same ID contained exactly the same individuals.

**Figure 1:**
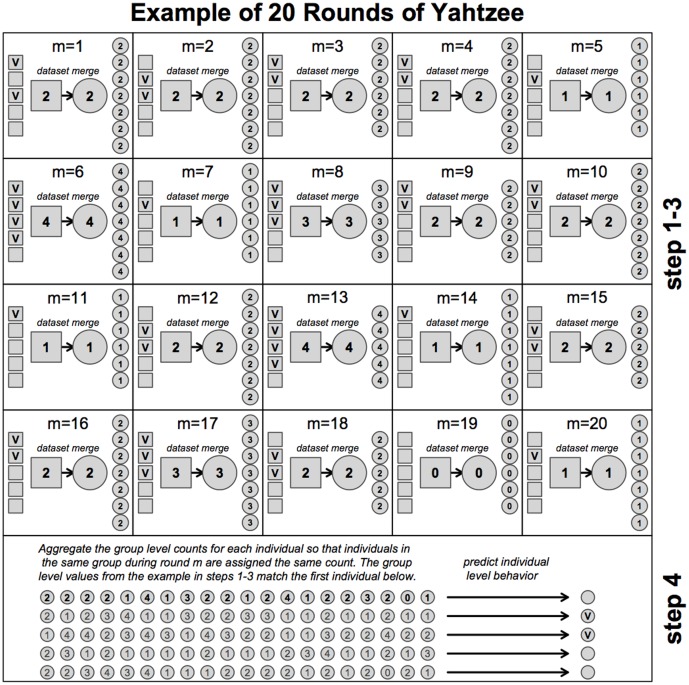
Step 1 of the Yahtzee procedure begins with the hashing of the datasets using a new salt (See Table 1 above). In step 2 the group ID is determined for all groups where 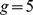 in the origin dataset and then matched to the same group ID from the destination group-level dataset. Notice that the hashing procedure and group aggregation is the same in both datsets except we keep all groups in the destination dataset, regardless of size. This is so because we only need to know the group size from the origin dataset to make predictions about the behavior in the destination dataset. Once the group-level datasets are matched by the group ID, the group-level information is stored and the process is repeated 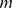 times. In step 3 the group level data is sent to the holder of the destination dataset so that the group level values can be assigned to the individual observations based on the same hashes used in the construction of the groups during each of the Yahtzee rounds. Once the destination dataset has acquired a sufficient number of group level values (see Figure 2 for information on determining the value of 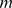) it is possible to then use the combined information to predict the behavior of each individual, which is step 4 of the Yahtzee procedure. For our application, using equations 4, 5 and 6 above, it is possible to predict if the individual is unregistered, a voter or an abstainer. Finally, it is worth repeating that only the group-level data is passed from the origin to the destination dataset. See the Pseudocode for additional information.

To make statistical inferences possible at an individual level, repetition of the grouping procedure is conducted 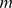 times, re-hashing using different seeds (and thus re-grouping individuals), and assigning an additional value to each user after every round. This repetition gave rise to our nickname for the procedure, “Yahtzee,” which refers to the idea of metaphorically re-rolling the dice on each iteration to place users in new groups. Figure 1 displays this process graphically.

Each respondent is assigned a distribution of 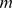 values, from the set 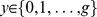. If 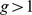 then it is not possible to infer with certainty the behavior of any individual user. However, given the distribution of these values, each additional draw provides more information about an individual's behavior. It is therefore possible to determine the 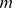 number of times the procedure should be repeated such that enough observations per person are recorded to classify an individual in the second dataset as behaving in a certain way. We describe how to calculate the estimate of the individual-level behavior in the next section and the number of 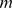 iterations necessary as we apply the method to data.

### Application of the Method

Recall that a “match” is defined as a row in both files that has the same values across files for ALL of the following columns in both the voter registration files (the origin dataset) and the Facebook data (the destination dataset): first_name, last_name, birth_day, birth_month, birth_year. Again, duplicate rows in both files are thrown out before any matching begins. Approximately 0.5% of Facebook users and approximately 0.5% of voters from the voter files were dropped due to duplication.

We conducted the procedure on each of several voter registration datasets. Facebook, as the holder of the larger dataset, then hashed the user record data (with the same sequence of random seeds and salts that we used), using first_name, last_name, and birth_day, birth_month, birth_year (also dropping duplicates) for users who logged in from the state in question on Election Day. An issue arose because the Facebook data does not ask users to explicitly name their first_name and last_name columns, but it does have a name column that contains the name provided by the user at time of registration. We defined first_name as the first token in name and last_name as the last token in the “name” field. This works well because most people enter their name such as “First M. Last”. However, it does not work if the name is entered as “The Illustrious First M. Last, Esquire,” which occasionally happens online. This inconsistency between datasets actually works to the advantage of those interested in the privacy preserving features of the method since individuals with names that do not follow the “First M. Last” are not matched and therefore add noise to the estimated individual-level values.

Facebook then divided the hash value derived from each name and birthdate by 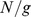 in order to create a group ID (note that 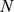 still represents the number of individuals from the origin dataset—i.e., the individual *public voter records*— not the number of individuals Facebook recorded as logging in from that state). This procedure is identical to the procedure used to create group IDs using public voting records. Therefore, individuals with the same first name, last name, and date of birth in both the public voting records and Facebook's data were assigned the same group ID.

This procedure guaranteed that any and all Facebook users who were also registered voters would be assigned the same group ID in both sets of data. However, because there was no guarantee that a given registered voter would also be a Facebook user, nor that a given Facebook user would be registered to vote (and therefore in the voter record), this procedure prevented identification of a specific Facebook user's behavior. The proportion of truly matched Facebook users in any group was unknown and could range from 0% to 100%.

Using the state voting data, we calculated the number of registered voters in each group who did vote (some number between 0 and 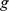) in 2010. We recorded that number and assigned it to each Facebook user with the same group ID. Importantly, a Facebook user was assigned to a group whether or not they were on the registration list. The group may have had any number of voters. So, in a given instance a user who was not on the registration list may be assigned to a group in which any fraction of those on the registration list voted. This feature of the procedure ensures that we cannot be certain that a particular Facebook user registered or voted based on the turnout value of their assigned group.

After repeating the Yahtzee process, each user was assigned a distribution of 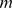 values, from the set 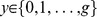. Because 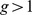, it is not possible to infer with certainty the voting behavior of any users, or even their registration status. As we describe above, each additional draw provides more information and we can set 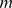 such that we have enough observations per person to classify individuals on Facebook as matched voters or matched abstainers with a minimum pre-determined level of measurement error (we chose a value of 5%).

To see why, notice that Facebook users who were not registered to vote would have an effectively random classification in every round. They are also randomly assigned to groups that have a random number of voters and abstainers in them. Therefore, if 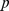 is equal to the turnout rate, then the probability that the 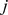
th draw for user 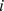 is equal to 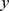 can be determined from a binomial distribution:
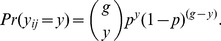
(1)


Meanwhile, users who were registered to vote would be somewhat more likely to have the correct classification (voter or abstainer). Given that the user was on the registration list, their presence in their own group in each draw skews the distribution of their own draws (toward 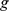 for voters and toward 0 for abstainers).

Specifically, if a Facebook record does match a voter record, then its own contribution to the total number of voters in the group is always 1, and since the other 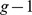 group members are randomly assigned, the probability that a draw is equal to 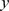 is
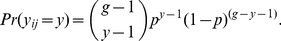
(2)


By the same reasoning, if a Facebook record matches an abstainer record, then its own contribution to the total number of voters in the group is always 0, and the probability that a draw is equal to 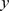 is simply
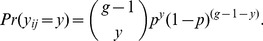
(3)


Since these are independent draws, the probability of observing the set of draws 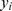 conditional on being unregistered, a voter, or an abstainer is
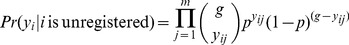
(4)

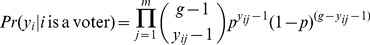
(5)

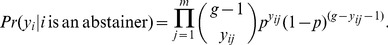
(6)


We can use these probabilities to classify individuals, assigning each to the classification that maximizes the likelihood of observing 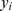. For improved efficiency we transform the equations to log likelihoods, and we use simulations to estimate the number of values needed per record (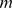) to generate a specific classification error. Simulation code (written in R) is provided in the Materials and Methods section.

### Selecting the Number of 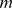 Iterations

For any application, we must select two values of 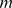 for each set of records that we wish to match in order to balance the rate of false voters and false abstainers. This is because the overall turnout rate determines which behavior takes fewer observations to distinguish from average behavior. If most people abstained, it will take fewer observations to identify groups where users likely voted, and vice versa. We therefore must make additional draws for individuals classified as belonging to the more frequent group. To achieve balanced rates we select two values: 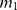 is the number of draws necessary to reach the desired level of accuracy for the less frequent behavior and 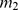 is the number of additional draws necessary to reach the desired level of accuracy for all individuals classified with the more frequent behavior (after 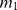 draws).

Choosing 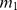 and 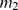 requires knowledge of the aggregate turnout rate, which was computed directly from the voter record. It also requires knowledge of the match rate (the probability a given Facebook record can be matched to a specific voter record). Therefore, for each state, Facebook estimated the match rate by drawing 1000 records at random from their database, and counting the number of matches with a list of the names and birth dates that were available in the voter record. No individual match was recorded: Only the aggregate match rate was stored, and all other information was discarded.

**Figure 2:**
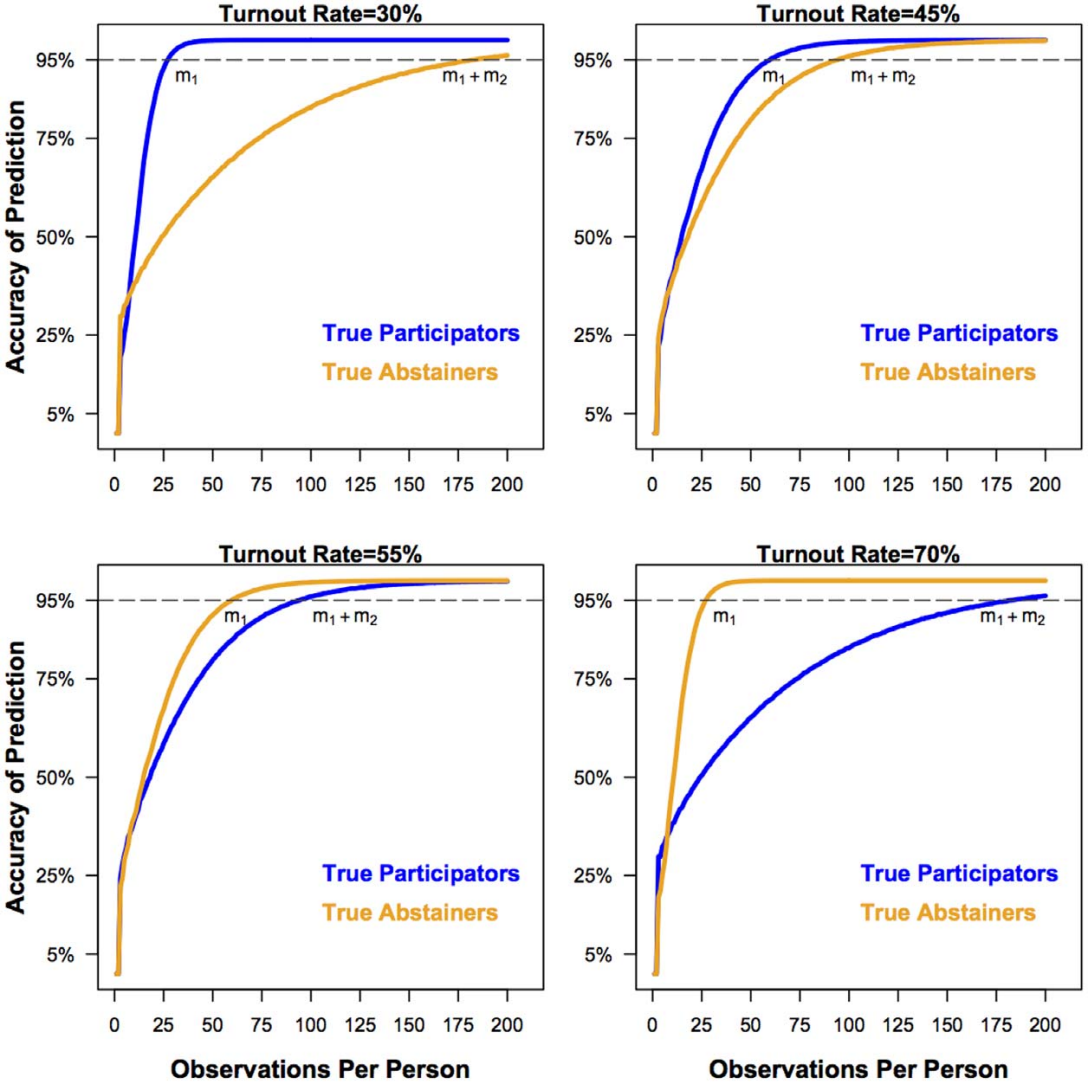
The proportion of correct predictions for participation rates of 30%, 45%, 55%, and 70% (the match rate is held constant at 30% in all four figures) from a simulation of the matching procedure. The dark line represents the accuracy rate for true participators. The light line represents the accuracy rate for true abstainers. Accuracy increases for both categories as observations for each individual are obtained from the Yahtzee procedure. Note that the less frequent of the two behaviors requires fewer observations for classification than the more frequent behavior. 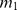 is the number of observations per person necessary to achieve a given level of accuracy for the less frequent behavior and 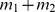 is the number of observations necessary to achieve a given level of accuracy for the more frequent behavior.

In order to test the method, we simulated the matching procedure using a set match rate that approximates what we observed in the 13 states that we used to match voter data (30%). We also set the turnout level to match each state in order to assess the prediction error associated with a given number of draws. The results of these simulations are summarized by Figure 2. The simulations show that the less frequent behavior necessitates fewer observations to classify individuals with a given level of confidence, and that as the turnout moves away from 50%, more observations (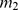) are needed to reach the level of confidence of the less frequent behavior.

It is important to note, once again, that the procedure only gives us 1) estimates of the probability that any given Facebook user is on the registration list and 2) estimates of his or her voting behavior. We can not be certain whether a user is on the list, has voted, or has abstained from these draws. In fact, it is possible that a voter will be misclassified as an abstainer, or that an abstainer will be misclassified as a voter. The number of draws is chosen such that classifications of this type are unlikely, but still possible. Using 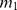 and 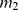 we are able to predetermine the measurement error level (in our case 95%) that appropriately balances the capability of inference and the protection of privacy of users. In other research applications, a higher or lower level of measurement error may be desired, which can easily be achieved by adjusting 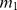 and 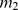 accordingly.

### Pseudocode

The following pseudocode summarizes the procedure. We also provide R code that can be modified to implement the procedure with any dataset. This code is available with the Supplementary Material S1.

**Pseudocode to process Origin Dataset.**

SET seed

CALCULATE a vector of salts

SAVE vector of salts

READ origin dataset

WHILE completed_observations < required_observations

- CONCATENATE record IDs from origin dataset with new salt
- CALCULATE a hash for each concatenated record ID
- CALCULATE modulus by dividing the hash value for each record by the value N/g
- SAVE modulus as the group ID
- CALCULATE the total number of records per group ID
- CALCULATE the number of records who exhibit a behavior per group ID
- CALCULATE the behavioral frequency for all groups that are of the specified group size (g = 5)
- SAVE group ID and behavioral frequency

END WHILE

#### Pseudocode to Process Destination Dataset

SET seed

READ destination dataset

READ vector of salts

READ group ID and behavioral frequency

WHILE completed_observations < required_observations

- CONCATENATE record IDs from destination dataset with new salt
- CALCULATE a hash for each concatenated record ID
- CALCULATE modulus by dividing the hash value for each record by the value N/g
- SAVE modulus as the group ID
- MATCH behavioral frequency BY group ID

END WHILE

SET group level behavioral frequencies to individual records in the destination dataset CALCULATE probability of behavior for individual records using the group level frequencies

## Results

### Validation

Our process yielded 6,338,882 “matches” from the voter files to Facebook's data. This number reflects the fact that we obtained about 1/3 of all voter records in the U.S., and of those, about 1/3 matched to the 61 million users who logged into Facebook on Election Day. To validate the Yahtzee process, we compared its classifications for a small set of randomly chosen records for each state to the true voting behavior of those users. Table 2 contains the 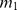 and 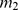 values for each state. These values were selected by adjusting the 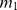 and 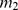 values in the simulation code in the Appendix until values yielding approximately 95% accuracy were found. That is, we decided that for our research purposes, it was appropriate to obtain 95% accuracy in estimating the likelihood that a particular individual in fact turned out to vote. Using the simulation code provided in the Appendix, we first adjusted 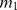 such that the algorithm would estimate with 95% accuracy the more common behavior in a particular state (either turning out to vote or abstaining). Given the 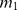 value, we then adjusted 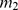 such that the less common behavior was also estimated with 95% accuracy.

**Table 2:** Number of Draws.

State	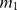	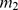	Common Type
Arkansas	55	45	Voters
California	50	50	Voters
Connecticut	65	10	Voters
Florida	75	0	Abstainers
Kansas	75	0	Abstainers
Kentucky	60	5	Voters
Missouri	70	20	Abstainers
New Jersey	60	65	Abstainers
Nevada	65	25	Voters
New York	55	50	Abstainers
Oklahoma	65	15	Abstainers
Pennsylvania	65	15	Voters
Rhode Island	75	5	Abstainers

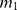
 is the number of draws necessary to reach the desired level of accuracy for the more frequent behavioral type and 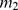 is the number of additional draws necessary to reach the desired level of accuracy for the less frequent behavioral type.

While implementing the algorithm on real data can be time consuming, determining the number of draws necessary to achieve a given level of accuracy is not. As seen in Table 2, the values for 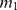 and 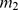 vary considerably. The variation in 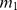 is due to variation in the turnout rate and variation in the match rate. A lower match rate requires more draws overall in order to distinguish those on the registration list from those not on the registration list. Variation in 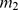 is primarily due to variation in the turnout rate in the states. States that have a turnout rate near 50% (such as Florida and Kansas) take few extra observations to distinguish the less common behavior from those who are assigned values at random, while states with a turnout rate far from 50% (such as Arkansas and New Jersey) require many extra draws to make such distinctions.

Table 3 shows conditional probabilities generated from truth tables for the Yahtzee classifier results. For each state, 1000 Facebook user records were chosen at random. Each was given a classification based on the Yahtzee process. The truth tables contained the frequency of each classification that was assigned to each true behavior. This information was used to calculate the classification accuracy in the categories of interest (voter or abstainer), which are displayed for each state in Table 3. We also calculated the 95% confidence interval for a null hypothesis that the prediction is correct 95% of the time (based on an assumption the successes are binomially distributed from the same number of draws observed). Note that nearly all of the confidence intervals contain the observed data, suggesting that deviations from 95% accuracy are due to sampling variation.

**Table 3:** Number of Draws.

State		Pr()	95% CI
	Pr(Abs 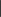 Class = Abs)	0.949	[0.908, 0.990]
Arkansas	Pr(Vot 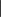 Class = Vot)	0.957	[0.913, 0.981]
	Pr(NM 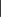 Class = NM)	0.988	
	Pr(Abs 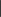 Class = Abs)	0.932	[0.912, 0.980]
California	Pr(Vot 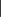 Class = Vot)	0.951	[0.919, 0.978]
	Pr(NM 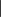 Class = NM)	0.987	
	Pr(Abs 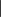 Class = Abs)	0.888	[0.916, 0.978]
Connecticut	Pr(Vot 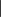 Class = Vot)	0.988	[0.912, 0.981]
	Pr(NM 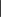 Class = NM)	0.994	
	Pr(Abs 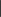 Class = Abs)	0.937	[0.914, 0.977]
Florida	Pr(Vot 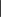 Class = Vot)	0.970	[0.917, 0.982]
	Pr(NM 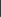 Class = NM)	0.998	
	Pr(Abs 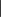 Class = Abs)	0.928	[0.915, 0.980]
Kansas	Pr(Vot 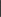 Class = Vot)	0.953	[0.918, 0.982]
	Pr(NM 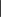 Class = NM)	0.996	
	Pr(Abs 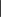 Class = Abs)	0.907	[0.921, 0.978]
Kentucky	Pr(Vot 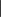 Class = Vot)	0.974	[0.921, 0.978]
	Pr(NM 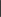 Class = NM)	0.993	
	Pr(Abs 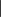 Class = Abs)	0.979	[0.915, 0.986]
Missouri	Pr(Vot 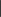 Class = Vot)	0.947	[0.904, 0.982]
	Pr(NM 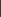 Class = NM)	0.999	
	Pr(Abs 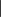 Class = Abs)	0.945	[0.908, 0.991]
New Jersey	Pr(Vot 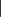 Class = Vot)	1.000	[0.895, 0.987]
	Pr(NM 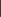 Class = NM)	0.999	
	Pr(Abs 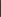 Class = Abs)	0.970	[0.917, 0.982]
New York	Pr(Vot 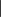 Class = Vot)	0.947	[0.908, 0.985]
	Pr(NM 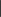 Class = NM)	0.987	
	Pr(Abs 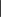 Class = Abs)	0.941	[0.911, 0.985]
Nevada	Pr(Vot 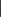 Class = Vot)	0.963	[0.915, 0.982]
	Pr(NM 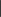 Class = NM)	0.996	
	Pr(Abs 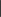 Class = Abs)	0.950	[0.914, 0.986]
Oklahoma	Pr(Vot 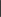 Class = Vot)	0.940	[0.920, 0.980]
	Pr(NM 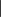 Class = NM)	0.998	
	Pr(Abs 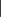 Class = Abs)	0.975	[0.912, 0.981]
Pennsylvania	Pr(Vot 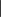 Class = Vot)	0.971	[0.914, 0.986]
	Pr(NM 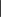 Class = NM)	0.994	
	Pr(Abs 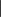 Class = Abs)	0.972	[0.908, 0.979]
Rhode Island	Pr(Vot 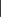 Class = Vot)	0.953	[0.912, 0.980]
	Pr(NM 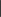 Class = NM)	0.997	

Yahtzee classifier results from 1000 randomly selected Facebook users from each state. Each user was given a classification based on the Yahtzee process: “Abs” 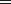 Abstainer, “Vot” 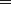 Voter, “NM” 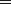 Not Matched. The conditional probabilities are calculated as the probability of observing a true behavior conditional on the Yahtzee classification. The 95% confidence intervals are for the null distribution of 95% accuracy in the classification, calculated from a binomial distribution with the same number of draws in each category. In total, 22 of the 26 tests fall within these intervals, suggesting that deviations from 95% accuracy are due to sampling variation, and for a large sample the procedure will generate the desired level of accuracy.

Researchers interested in this procedure have the ability to increase or decrease the accuracy of the group level matching procedure by increasing or decreasing the number of observations generated for each user. At the limit (extremely high values of 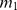 and/or 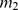) researchers have the ability to draw enough observations that they are extremely confident about the true behavior of users, but because of the group-level matching nature of the procedure, they will never be 100% certain of an individual's behavior.

In addition to estimates of the voting behavior of individuals, the procedure yields estimates of the probability that an individual is on the voting record at all. As Table 3 shows, there is approximately a 99% chance that when we do not find a match for a user that there was not a match for that individual on the state's voting record. However, a user was “matched” only when there was perfect concordance for first name, last name, and birthdate. While we might be nearly certain that there was no match for the user, the presence of nicknames, variation in reported birth date, and other errors in the data mean that unmatched users might actually be in the voter record. Thus, this confidence level represents an upper bound of the probability that a user is not in the record, given that we classified them as not matched is probably lower than 99%. This means that interpretations of analyses based on the unmatched classification should be careful to describe the process as measuring the match rate rather than measuring the exact likelihood that a given user was in the record. It also means that user privacy is more protected by uncertainty since there is a greater chance that the user was actually in the record when the procedure classifies him or her as unmatched. Thus, this confidence level represents an upper bound of the probability that a user is not in the record, given that we classified them as not matched is probably lower than 99%.

**Figure 3:**
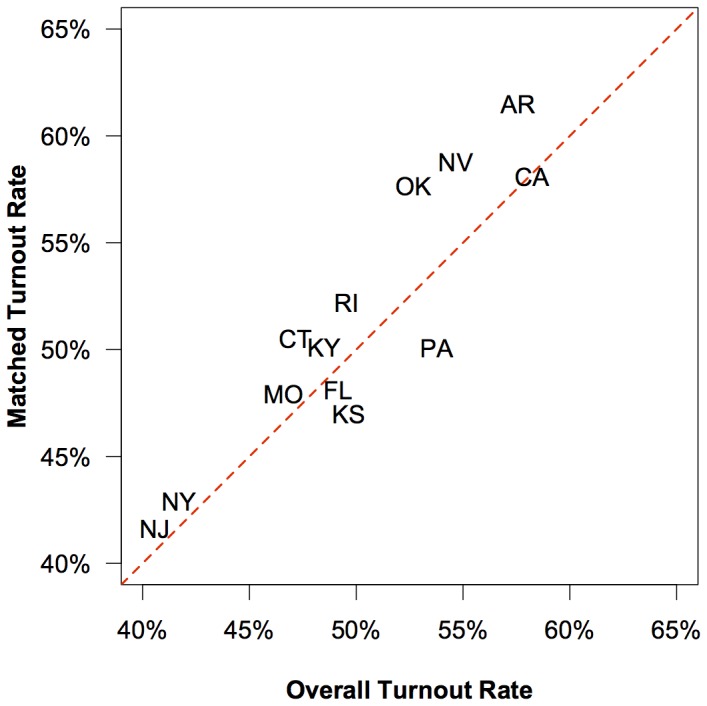
The proportion of matched users who turned out to vote compared to the overall turnout rate by state. Note that the abbreviation for Kansas is repositioned slightly so that it does not overlap with the abbreviation for Florida. The results show that the Yahtzee procedure produces about the same overall turnout rate for each state as that shown in the official voter record.

Although it is possible that there are important systematic differences between users with matchable and unmatchable records, we tested for some such differences and found little evidence to support this idea. Figure 3 shows that there is a good fit between the turnout rate of matched Facebook users and the overall turnout rate of each state. This positive relationship suggests that the matching procedure is producing reliable estimates of turnout for matched users at an aggregate level. However, the relationship is not perfect for at least two reasons. First, Facebook users within a given state are not necessarily a representative sample of that state's registered population. For example, we know that the age distribution of Facebook users skews toward younger people. Second, matched users are not necessarily representative of all Facebook users, including those who could not be matched. For example, people who use exotic nicknames may have personality traits that also affect their willingness to vote. Thus, while the good aggregate level fit is suggestive, we should be cautious when describing our results to explain the limitations of out-of-sample inferences that might be made using the matched data.

**Figure 4:**
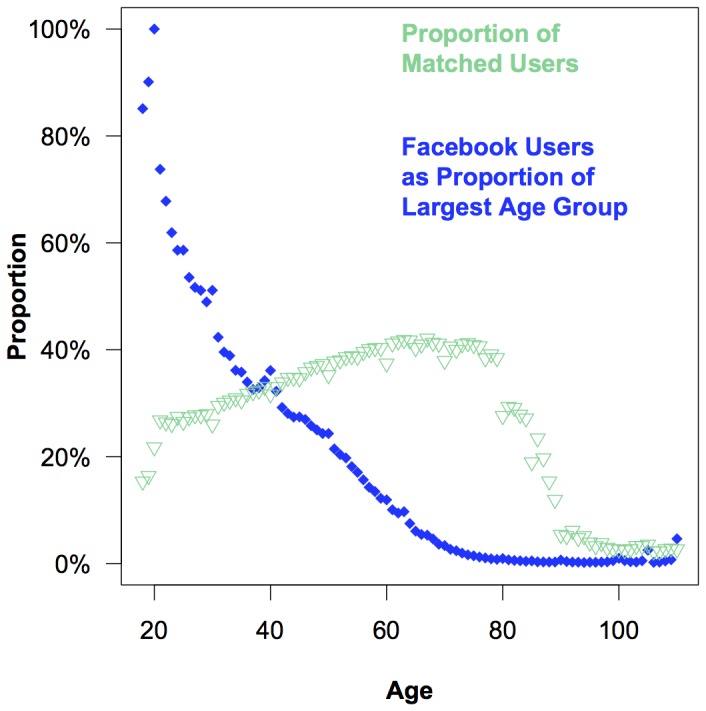
The proportion of Facebook users that were matched to the validated voting record by age and each age group's proportion of the largest age group (those 20 years of age at the time of the election). This figure helps to explain why match rates are lower for Facebook users who tend to be younger and more difficult to match than the average registered voter.

About one in three Facebook users were successfully matched to their state's voter records using the Yahtzee process. Although the match rate for this study is lower than the match rates in other studies that match individuals to public voting records (which typically atain match rates about 50%), this may be due to the demographic composition of Facebook. In Figure 4 we show how the probability of matching varies by age. There is a positive relationship between age and the probability of matching the voting record through approximately age 80 (as seen by the positive slope of the triangles). While there is a drop off in the probability of obtaining matches for users over the age of 80, it is important to note that there are very few Facebook users in this age group (as seen by the left skew of the diamonds). Younger users are also more difficult to match, likely because fewer of them are registered, and even those who are registered may be accessing Facebook from an out-of-state college. Older Facebook users are easier to match, but there are fewer of them.

**Figure 5:**
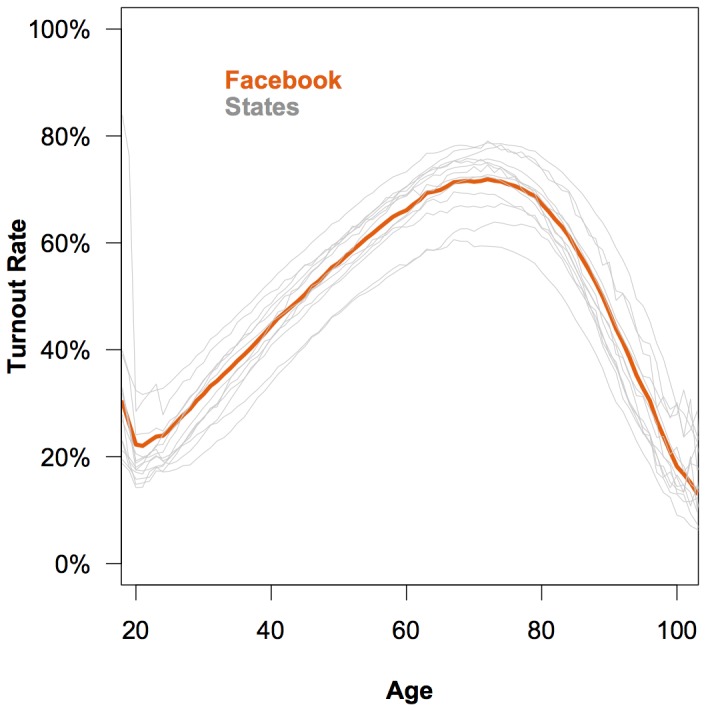
The proportion of matched users who turned out to vote by age. The dark line represents the turnout rate by age of the matched sample of Facebook users. Each gray line represents the turnout rate by age of a state voter record. The results show that users on Facebook exhibit the same pattern of turnout with respect to age as the populations of each state.

Because we know that the matched sample is not representative of the overall population by age, we assessed the turnout rate of the matched Facebook user sample as it compared to the turnout rate of each state by age. Figure 5 shows that the turnout rate goes up among older users and declines with advanced age in both the matched sample and the voter records for each state. These results suggest that the matching procedure correctly identifies voters and abstainers and that once we control for the skew in the age distribution of the matched sample, the voting behavior of Facebook users is not very different from that of the population overall.

### Friends and Voting

The Yahtzee procedure allowed us to combine several distinct public voting records with Facebook data while maintaining the anonymity of the users. An important question in the literature on voting, which we can now address with this data, is the extent to which voting behavior is correlated between socially-connected individuals [12]. Scholars have long known that turnout is strongly correlated between friends, family members and coworkers, even when controlling for socioeconomic status and selection effects [13]–[24]. Some of this correlation may result from the tendency to choose friends with a similar tendency to be engaged in politics (“homophily”), and some of it may result from a tendency for socially-connected individuals to affect each other's political behavior (“influence”), but both are important phenomena and establishing a correlation is the first step in determining whether either exists [25].

The Facebook data is particularly appealing for addressing correlated behavior because it also allows us to measure the *strength* of the social connection between two users. We expect that correlation in voting among closer friends should be higher because of several mechanisms or combinations of them. As mentioned above, people choose friends, and in particular close friends, based on similarity in attributes (“homophily”). If individuals are selecting friends based on a shared interest in politics or civic activism then they are more likely to both vote because of the shared interest. Closer friends are also more likely to “influence” each others' behavior [11]. A friend might observe the other friend voting and then vote herself, especially if they carpool or work together. Also, one friend could discuss an upcoming election with another friend and convince or remind her to vote. Finally, closer friends are more likely to be physically proximate [26], and thus be more likely to both be exposed to the same environmental factors. For instance, close friends may be more likely to live in the same competitive district or both be exposed to the same “get out the vote” drive. There are many possible scenarios that could lead to a correlation between friendship strength and voting behavior.

In order to determine friendship strength of the users in our sample, we followed the recommendations of [27] and created a measure based on the interactions between two users. Interactions include actions on Facebook that could be directed from one user to another and include: *comment*, *like*, *message*, *poke*, *wall post*, *tag* or *chat*. As described in [11], we categorized all friendships in our sample by decile, ranking them from lowest to highest percent of interactions. Each decile is a separate sample of friendship dyads. For example, decile 1 contains all friends at the 0th percentile of interaction to the 10th percentile while decile 2 contains all friends at the 11th percentile of interaction to the 20th, and so on. We validated this measure of tie strength with a survey [11], [27]. In this survey we asked Facebook users to identify their closest friends. Subjects were randomly asked to identify either 1, 3, 5, or 10 friends We then measured the percentile of interaction between friends in the same way and predicted survey response based on interaction. The results show that as the decile of interaction increases, the probability that a friendship is with the user's closest friend increases. This finding is consistent with the hypothesis that the closer a social tie between two people, the more frequently they will interact, regardless of medium. In this case, frequency of Facebook interaction is a good predictor of being named a close friend. Moreover, previous research suggests that higher levels of interaction on Facebook indicate that such friends are more likely to be physically proximate and suggest a higher level of commitment to the friendship, more positive affect between the friends, and a desire for the friendship to be socially recognized [26].

With the matched data and a measure of friendship strength we now have the information necessary to test the relationship between friendship strength and voting behavior with the following hypothesis:

#### Hypothesis

Similarity in voting behavior between socially-connected individuals increases as the strength of their relationship increases.

**Figure 6:**
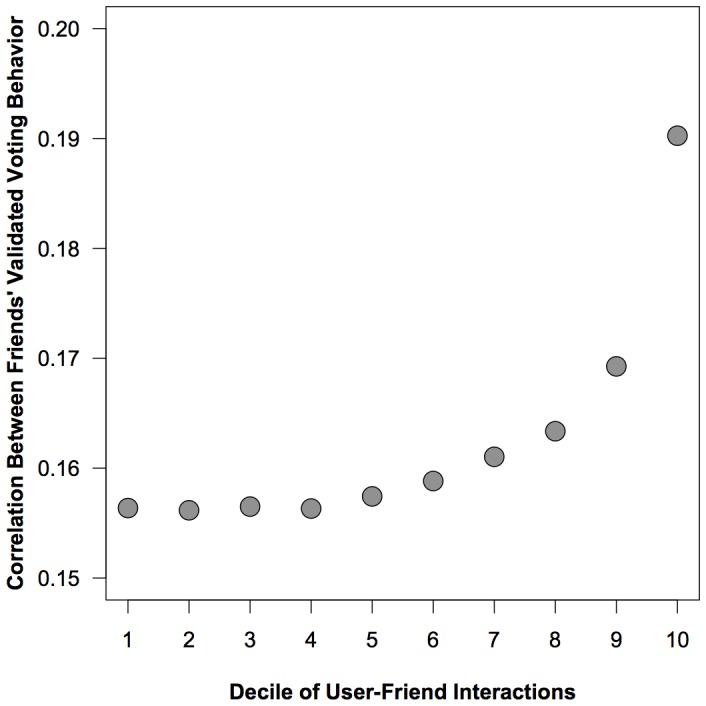
The correlation between friends' validated voting behavior based on the proportion of interaction between the dyad in the three months prior to the election. We categorized all friendships in our sample by decile, ranking them from lowest to highest percent of interactions. Each decile is a separate sample of friendship dyads. For example, decile 1 contains all friends at the 0th percentile of interaction to the 10th percentile while decile 2 contains all friends at the 11th percentile of interaction to the 20th, and so on. Interactions include actions on Facebook that could be directed from one user to another and include: comment, like, message, poke, wall post, tag or chat. These correlations exist well outside of simulated null distributions. 95% confidence intervals are displayed in Table 4.

To test this hypothesis we used the validated decile measure of tie strength and then calculated the correlation between user and friend's validated voting behavior for all of the friendship dyads (see Figure 6). The correlation in friends' validated voting behavior increases as tie strength increases, suggesting that closer friends have more similar voting behavior than more distant friends.

However, we want to determine if the correlations we observe are different from what we would expect due to chance. Standard techniques assume independence of observations, which is not the case here due to the complex interdependencies in the network. To take the network into account, we compare the observed correlation to a randomly-generated value when we keep the network topology fixed but randomly permute the voting behavior of friends and once again measure the correlation. We repeat this procedure 1,000 times to generate a theoretical null distribution for the correlation we would expect due to chance. We obtain confidence intervals for the null distribution by sorting the results and taking the appropriate percentiles (in our case, we are interested in the 95% confidence interval, so we use the 25th and 975th values). These low and high values are displayed in Table 4. The results show that all of the observed correlations are well outside of the 95% confidence intervals of the null distributions. The narrow range of variation in the null distribution also suggests that the behavior of the closest friends is significantly more correlated than average.

**Table 4:** Correlation.

Decile	Correlation	n	Null low	Null high
1	0.156366	6703469	−0.000960	0.000976
2	0.156163	7750717	−0.000905	0.000815
3	0.156499	8253044	−0.000812	0.000840
4	0.156321	8552987	−0.000766	0.000832
5	0.157428	8882969	−0.000750	0.000809
6	0.158828	8847712	−0.000815	0.000793
7	0.161030	9041679	−0.000794	0.000709
8	0.163359	9407243	−0.000736	0.000755
9	0.169258	9356494	−0.000788	0.000762
10	0.190253	9122388	−0.000761	0.000785

The estimated correlation between a user's validated turnout and the validated turnout of her friends by decile of user friend interactions. See Figure 5 for a visualization of this relationship. To compare the observed values to what is possible due to chance, we keep the network topology fixed and then randomly permute the voting behavior of friends. We repeat this procedure 1,000 times and measure the correlation. The simulated correlation values generate a theoretical null distribution for the correlation which we would expect due to chance. The Null low and Null high columns display the 95% confidence interval of this null distribution. Note that the observed correlations exist well outside the null distributions.

The Yahtzee procedure has allowed us to repeatedly match small groups of anonymous individuals between datasets without ever sharing individual level information between the datasets. The method allowed us to ensure the anonymity of the user by adding uncertainty about each individual's behavior in the dataset. The method is conceptually similar but procedurally distinct from other techniques designed to ensure respondent anonymity at the time of data collection [4]–[7]. We have demonstrated that the method performs as predicted by theoretical analysis by applying it to data from Facebook and public voter records. The procedure allowed us to demonstrate that individual-level users' validated voting behavior is correlated with the behavior of the user's closest friends. This inference was made using data from two distinct sources that were never combined at the individual level.

## Discussion

Here we have introduced a method of group-level matching that allows researchers to merge two data sources while respecting the privacy of individuals in the constituent data sets. Methods like these are essential for researchers who are interested in making inferences that draw upon data about individuals, while also respecting the privacy of individuals (and the privacy policies of entities that collect such data). Many extraordinary research projects could be enhanced by joining their data with other sources of individual information. For example, one project collected 509 million Twitter messages from 2.4 million individuals from 84 countries between February 2008 and January 2010 [28]. Other studies include an analysis of the mood within America as a function of date and time using 300 million Twitter messages generated between September 2006 and August 2009 [29], an analysis of 50 million Google search queries to identify the weekly influenza level in regions of the United States [30], and an analysis of the application adoption patterns of 50 million Facebook users [31]. In each of these studies, however, a variety of additional questions could be addressed if more information about the users generating the observed data could be obtained. This information often exists in other datasets, yet linking these datasets raises both technical and ethical concerns.

We used the Yahtzee method to match public voting records to Facebook user data. Though our application focuses on voting behavior, other respondent behaviors and outcomes — such as data found in medical reports or consumer records — could be matched to other data sources using the Yahtzee method as well.

Our voting behavior application allowed us to test the Yahtzee method on real world data, where we found that it generates the same level of uncertainty about individual records that was predicted by theory. Additionally, we found that the turnout rate of Facebook users by state strongly correlates with the overall turnout rate of all individuals in the state and Facebook users within each age group tend to vote at about the same rate as members of those age groups in the population as a whole. These results not only suggest that the Yahtzee method works as expected, but also that Facebook users are very similar to the population as a whole in terms of their voting behavior. This should be an encouraging result for a growing group of researchers who rely on Internet websites such as Facebook or Amazon's Mechanical Turk in order to recruit subjects. Finally, we showed that using this data we are able to study the correlation in voting behavior between friends, finding that the correlation in behavior becomes tighter as friendships become closer.

We live in an age in which more and more data are being collected about individuals, providing researchers with the opportunities to study phenomena at a scale never before possible and to study new relationships that were previously infeasible to measure due to the difficulty of collecting information from diverse sources about the same individuals. While the availability of this data offers exciting opportunities for new avenues of research, much of it is held by corporations that have an interest in maintaining the privacy of their users or customers. In order for researchers to conduct studies using this data, new methods will need to be invented that fit specific problems with the data. In this paper, we offer one solution to what we believe is a common problem that corporations and researchers often face: the need to ethically and respectfully match sensitive individual-level data to additional sources of information.

## Supporting Information

Supplementary Material S1 R program that can be modified to implement the procedure with any dataset. R program that estimates the number of values needed per record to generate a specific classification error.
(ZIP)
